# Identification and Functional Testing of Novel Interacting Protein Partners for the Stress Sensors Wsc1p and Mid2p of *Saccharomyces cerevisiae*

**DOI:** 10.1534/g3.118.200985

**Published:** 2019-02-07

**Authors:** Ednalise Santiago-Cartagena, Sahily González-Crespo, Vladimir Vélez, Nelson Martínez, Jamie Snider, Matthew Jessulat, Hiroyuki Aoki, Zoran Minic, Pearl Akamine, Inoushka Mejías, Luis M. Pérez, Brian C. Rymond, Mohan Babu, Igor Stagljar, José R. Rodríguez-Medina

**Affiliations:** * Department of Biochemistry, University of Puerto Rico, Medical Sciences Campus, PO Box 365067, San Juan, PR 00936-5067; † Donnelly Centre, Department of Biochemistry, Department of Molecular Genetics, University of Toronto, Ontario M5S 3E1, Canada; ‡ Department of Biochemistry, University of Regina, Regina, Saskatchewan S4S 0A2, Canada; § Molecular Sciences and Research Center, University of Puerto Rico, 1390 Ponce de Leon Avenue, Suite 1-7, San Juan, Puerto Rico 00926; ** Department of Biology, University of Kentucky, Lexington, KY 40506; †† Mediterranean Institute for Life Sciences, 21000 Split, Croatia

**Keywords:** cell wall, stress response, drug resistance, oxidative stress, *Saccharomyces cerevisiae*

## Abstract

Wsc1p and Mid2p are transmembrane signaling proteins of cell wall stress in the budding yeast *Saccharomyces cerevisiae*. When an environmental stress compromises cell wall integrity, they activate a cell response through the Cell Wall Integrity (CWI) pathway. Studies have shown that the cytoplasmic domain of Wsc1p initiates the CWI signaling cascade by interacting with Rom2p, a Rho1-GDP-GTP exchange factor. Binding of Rom2p to the cytoplasmic tail of Wsc1p requires dephosphorylation of specific serine residues but the mechanism by which the sensor is dephosphorylated and how it subsequently interacts with Rom2p remains unclear. We hypothesize that Wsc1p and Mid2p must be physically associated with interacting proteins other than Rom2p that facilitate its interaction and regulate the activation of CWI pathway. To address this, a cDNA plasmid library of yeast proteins was expressed in bait strains bearing membrane yeast two-hybrid (MYTH) reporter modules of Wsc1p and Mid2p, and their interacting preys were recovered and sequenced. 14 previously unreported interactors were confirmed for Wsc1p and 29 for Mid2p. The interactors’ functionality were assessed by cell growth assays and CWI pathway activation by western blot analysis of Slt2p/Mpk1p phosphorylation in null mutants of each interactor under defined stress conditions. The susceptibility of these strains to different stresses were tested against antifungal agents and chemicals. This study reports important novel protein interactions of Wsc1p and Mid2p that are associated with the cellular response to oxidative stress induced by Hydrogen Peroxide and cell wall stress induced by Caspofungin.

The frequency of invasive mycoses due to opportunistic fungal pathogens has increased significantly over the past two decades (Rees *et al.* 1998; Trick *et al.* 2002; Ostrosky-Zeichner *et al.* 2003; Hajjeh *et al.* 2004; Pfaller and Diekema 2004; Pfaller *et al.* 2004b, 2004a; Walsh *et al.* 2004). In patients with health conditions that weaken the immune system or in those who may be predisposed to invasive fungal infections in intensive care wards, opportunistic infections with *Candida albicans*, *Aspergillus fumigatus*, *Cryptococcus neoformans* or other common fungal pathogens can have mortal consequences (McNeil *et al.* 2001; Yoon *et al.* 2014). The arsenal of therapeutic antifungal drugs currently in use, which includes echinocandins that target cell wall synthesis, polyenes that interact with sterol and forms channels in the plasma membrane, azoles that target sterol synthesis, and pyrimidine analogs, a more recent drug class that targets DNA synthesis, is relatively limited compared to the wide range of antibiotics available against bacterial pathogens (Scorzoni *et al.* 2017). As fungi share similarities in metabolic pathways with their mammalian hosts, the search for novel drug targets that are uniquely expressed in fungi is a fundamental requirement for development of non-toxic antifungal drugs. Therefore, it is imperative to investigate the mechanisms employed by fungi to overcome stress provoked by factors that challenge their cellular integrity. In *Saccharomyces cerevisiae*, the Protein Kinase C (Pkc1p) activates a mitogen-activated protein kinase (MAPK) cascade that phosphorylates the protein kinase Slt2p/Mpk1p in the cell wall integrity (CWI) pathway (Heinisch *et al.* 1999). In *S. cerevisiae*, activation of the CWI pathway is often monitored by assessing the phosphorylation status of Slt2p (Mpk1p), where phosphorylated Slt2p is the readout for Pkc1p activation.

CWI pathway participates in the regulation of cell wall biosynthesis and maintenance of cell integrity (Zarzov *et al.* 1996; Verna *et al.* 1997). In *S. cerevisiae*, this pathway is implicated in the response to a wide variety of stresses, including heat-shock (Kamada *et al.* 1995), hypo-osmotic shock (Davenport *et al.* 1995), nutritional stress (Torres *et al.* 2002), impaired cell wall synthesis (Ketela *et al.* 1999), antifungal drug treatments, and other environmental stresses that can alter the integrity of the cell wall (Vilella *et al.* 2005). The CWI pathway is well conserved among fungi. The Slt2p homolog in *C. albicans* (Mkc1p), and in *C. neoformans* (Mpk1p), are required for maintenance of the cell wall integrity and cell fitness at high temperature (Navarro-García *et al.* 1995; Kraus *et al.* 2003; Zhao *et al.* 2007). Likewise Pkc1p, a fundamental component of the CWI pathway, is also conserved between *Candida albicans*, *Cryptococcus neoformans* and *Aspergillus fumigatus* (Heinisch and Rodicio 2018).

In *S. cerevisiae*, cell wall perturbations are sensed by transmembrane sensors of the *WSC* family (Wsc1p, Wsc2p and Wsc3p), as well as Mid2p and its homolog Mtl1p (Gray *et al.* 1997; Verna *et al.* 1997; Ketela *et al.* 1999; Green *et al.* 2003). All the sensors share a signal peptide, a predicted type I transmembrane domain, a relatively short cytoplasmic tail ranging from 92 amino acids (for Wsc1p) to 120 (for Mid2p), and an extracellular domain with sequences rich in serine and threonine that are highly O-mannosylated (Verna *et al.* 1997; Philip and Levin 2001; Lommel *et al.* 2004; Rodicio and Heinisch 2010). A difference between these proteins is that the *WSC* family members have a cysteine-rich motif near the N-terminus (Verna *et al.* 1997), while Mid2p and Mtl1p have a single high mannose N-linked glycan in their N-terminus (Hutzler *et al.* 2008; Bermejo *et al.* 2010). Despite this difference, both N-terminal regions are important for receptor-specific sensing of cell wall damage. Additionally, the C-terminus of Mid2p has an aspartic acid residue region that has been suggested to resemble a Ca^2+^-binding domain and is important for the mating pheromone process (Ono *et al.* 1994; Vilella *et al.* 2005). The relative abundances of the five transmembrane sensors are quite variable. According to Kulak *et al.* (2014), on average, unstressed cells of *S. cerevisiae* have approximately 271 molecules of Wsc1p, 24 molecules of Wsc2p, 301 molecules of Mid2p, and 11 molecules of Mtl1p per cell, while the number of Wsc3p molecules has not been determined. The Wsc1 protein is known to form patches on the cell surface (Rodicio and Heinisch 2010) and specifically the cysteine-rich domain is involved in the clustering and homodimeric interactions of this sensor (Kock *et al.* 2016). In turn, this clustering is needed for CWI signaling to occur (Straede and Heinisch 2007). Protein-protein interactions (PPIs) at the cytoplasmic tails of the sensors should also be required to transmit these extracellular signals to effector proteins inside the cell. It has been suggested that the most important cell wall sensors for the response to cell wall stress are Wsc1p and Mid2p, with Wsc2p and Wsc3p reportedly having functions that are redundant with that of Wsc1p (Verna *et al.* 1997; Ketela *et al.* 1999). Wsc1p and Mid2p share complementary essential function(s) as evidenced by the lethality of a *wsc1∆mid2∆* double deletion mutant strain. In addition, they respond similarly with respect to thermal stress. However, they have been found to respond differently to other environmental stimuli. For example, Mid2p has been associated with CWI pathway activation in response to Calcofluor white (Ketela *et al.* 1999; de Nobel *et al.* 2000), mating pheromone (Errede *et al.* 1995), vanadate (Martín *et al.* 2000), and acidic conditions (Claret *et al.* 2005), while Wsc1p has been associated with activation of this pathway in response to Caspofungin (Reinoso-Martín *et al.* 2003), alkaline pH (Serrano *et al.* 2006) and reorganization of actin during hypo-osmotic stress (Gualtieri *et al.* 2004). On the other hand, the *WSC2* and *WSC3* genes act as multi-copy suppressors in mutants with glycerol synthesis abnormalities (Wojda *et al.* 2007; Rodicio and Heinisch 2010). Mtl1p has been associated with the response to hydrogen peroxide-induced oxidative stress and glucose deprivation (Vilella *et al.* 2005; Petkova *et al.* 2010). Wsc1p and Mid2p orthologs have been identified in *Kluyveromyces lactis* (Rodicio *et al.* 2008), *Aspergillus fumigatus* (Dichtl *et al.* 2012) and other yeasts, indicating that they may share similar signaling mechanisms. Further homology analysis revealed that no human homolog for Mid2p is described and Wsc1p shared 2% of protein query coverage with human mucin-15 isoforms b or c, and no significant similarity was found with mucin-15 isoform a, indicating that Wsc1p and Mid2p are potentially useful drug targets.

Evidence for Wsc1p and Mid2p signaling directly through Rom2p, a Rho1p GEF, was first provided by yeast two-hybrid data using only the cytoplasmic tail of each sensor (Philip and Levin 2001). Rom2p attaches to the cytoplasmic tail of Wsc1p and Mid2p in a regulated manner as the initial step that eventually leads to the activation of Pkc1p by the small GTPase Rho1p (Philip and Levin 2001). In the case of Mid2p, it has been reported to activate the Pkc1 pathway in a Rom2p-independent manner by interacting with Zeo1p, which acts as an adaptor protein (Green *et al.* 2003). The interaction of the Wsc1p with Rom2p requires dephosphorylation of serine residues in the cytoplasmic tail of Wsc1p. It is unclear how these phosphorylations are regulated, what proteins participate in the phosphorylation reaction, and if there are other adaptor proteins like Zeo1p that regulate the Rom2p interaction. To identify additional components of this signaling pathway that can lead to a better understanding of how the signaling mechanism is regulated during vegetative growth, we set out to expand the interactomes of Wsc1p and Mid2p in steady state (no stress) growth conditions. For this we used the integrated Membrane Yeast Two Hybrid (iMYTH) method, a robust technique that has been valuable for the identification of protein-protein interactions (PPIs) involving membrane proteins (Paumi *et al.* 2007, 2008; Snider *et al.* 2013; Lam *et al.* 2015; Santiago *et al.* 2016; Sokolina *et al.* 2017).

## Materials and Methods

### Strains, transformations and growth conditions

To create iMYTH bait strains, iMYTH L2 or L3 cassettes containing the 35-40 bases of homology near the C-terminal end of the *WSC1* or *MID2* gene (minus the stop codon), C-terminal fragment of ubiquitin, yellow fluorescence protein (L3 cassette only) and transcription factors [C_ub_- (YFP)-LexA-VP16 KanMX] (Snider *et al.* 2010) were amplified by PCR and transformed into MYTH reporter strains (THY.AP4 or L40) and selected on YPAD solid medium containing G418 (200 μg/ml). The genomic DNA of the bait strain was used to verify the insertion of the cassette by PCR using a forward primer internal to the *WSC1* or *MID2* genes and a reverse primer internal to the *KanMX* gene (Snider *et al.* 2010). The iMYTH bait validation and localization were performed as described in Snider *et al.* (2010).

The *wsc1*∆, *mid2∆*, and prey protein single deletion mutant strains (Open Biosystems) were generated by single gene replacement with a *KanMX4* module by homologous recombination using a PCR based strategy (Wach *et al.* 1994) in the BY4742 genetic background. Haploid strain *mid2*::*URA3 (mid2∆)* was obtained by sporulation of a heterozygous diploid strain (Thermo Scientific Open Biosystems). The wild type strains, BY4741 and BY4742, were obtained from ATCC. See Table S1 for yeast strains and Table S2 for DNA oligonucleotides used in this study.

### Library screening

Library screening and transformation was conducted as described by Snider *et al.* (2010). The culture for the transformation were grown at 30° or 37° and the cells were plated onto BD-Trays containing SD-WAH or SD-WH and incubated at 30° or 37° for 3-4 days. For the Optimized Large Scale Transformation, 20 ml instead of 200 ml 2x YPAD of bait strain culture was transformed with 40-80 μg of N_ub_G-X cDNA prey library.

### Plasmid recovery and sequencing

Single colonies obtained from the large-scale iMYTH transformation of bait strains were picked and diluted into 50 μl sterile 0.9% NaCl solution. Afterward, 2.5 μl of re-suspended cells were plated onto SD-WAH or SD-WH plates containing X-Gal and grown for 1-3 days at 30° or 37°. Then, blue colonies were inoculated into SD-W in 96-well blocks and grown for 2 days at 30° or 37°. The pellets were re-suspended with 125 μl of Lysis solution [β-mercaptoethanol, Solution A (1M Sorbitol, 0.1 M Sodium Citrate, 60 mM EDTA) and Zymolase Solution (Zymolase powder, 1 M Sorbitol)] and treated with Zymolase for 2 hr at 37°. The Nucleospin 96-well miniprep kits were used according to the manufacturer’s protocol.

Competent cells of *E. coli*, DH5α strain, were transformed with the plasmids recovered from yeast minipreps and plated on LB agar with 100 μg/ml ampicillin. For high-throughput transformation, 96-well plates and 96-well blocks were used. For *E. coli* minipreps, a 96-well block with 1.2 ml of Terrific Broth containing 100 μg/ml ampicillin was inoculated with a single colony in each well and grown for 2 days at 37°. The plasmids were purified using Nucleospin columns and the plasmids were sequenced using the N_ub_G forward internal oligonucleotide (Table S2). In-house software was used for large-scale BLAST analysis and identification of yeast protein sequences.

### Bait dependency test

The purified prey plasmids were transformed into the yeast bait strains Wsc1 THY L2, Mid2 THY L3, and A0286 or Wsc1 L40 L3, Mid2 L40 L3 and A0287. Note that A0286 and A0287 correspond to ‘negative control’ reporter strains stably expressing an artificial bait construct, in THY.AP4 and L40 backgrounds, respectively. The resulting transformations were plated into SD-W media and incubated at 30° or 37° for 3-4 days. The transformant colonies were selected in triplicate and plated onto SD-WAH + X-Gal or SD-WH + X-Gal. Preys that caused growth and blue coloration in a bait strain, but not in control bait strain, were considered to be specific or true interactors (Paumi *et al.* 2007; Snider *et al.* 2010). The specific interactors for the sensor proteins were classified according to their biological process. Cytoscape program (version 3.2.1) was used to generate the interactome maps (Figures 3 and S4).

### Monitoring CWI pathway under stress conditions

For thermal stress testing, 15 ml cultures of wild type and bait/prey null mutant strains were grown until OD_600_ readings ∼0.7-0.9 were reached. Cultures grown without heat stress conditions were conducted in 15 ml of media at 27° to which 15 ml of media at 27° were added and continued for 1 hr. Cultures grown under heat stress conditions were conducted in 15 ml of media at 27° to which 15ml of media pre-heated at 53° were added, mixed rapidly, and continued at 37° for 1 hr. For cell wall stress, 75 ng/ml of Caspofungin was added to 25 ml of culture at OD_600_ ∼0.7-0.9. For oxidative stress, Hydrogen peroxide was added at a final concentration of 1 mM to 25 ml of culture at OD_600_ ∼0.7-0.9. The cultures were compared with 25 ml of culture without stress. The cultures were incubated at 27° for 1 hr.

After the treatment, the cultures were centrifuged for 5 min at 4,800 rpm and the cells washed with ice cold CSM media and transferred to 1.5 ml microtubes. The cells were resuspended in ice cold CSM media centrifuged again in microtubes at 14,000 rpm at 4° for 3 min and resuspended in lysis buffer (50 mM Tris-HCl, pH 7.5, 10% glycerol, 1% TritonX- 100, 0.1% SDS, 150 mM NaCl, 5 mM EDTA) supplemented with 5X Protease Inhibitor Cocktail (Roche), 1X phosphatase inhibitor cocktail (II and III, Sigma) and 5X PMSF. The cells were finally disrupted by the addition of approximately 400 µl of glass beads to the tubes, after which the contents were subjected to five sets of alternate vortexing at full speed for 30 sec followed by 3 min incubation on ice. After disruption, the contents in the tubes were centrifuged at 13,000rpm for 10 min at 4° and the clarified protein supernatants were transferred to new pre-chilled 1.5 ml microtubes. Afterward, small portions of each extract were taken aside for determining protein concentration using the DC Protein Assay (Bio-Rad). The proteins in the remaining portions of the extracts were then denatured by heating at 95° in the presence of a 5X SDS solution and 5% per volume β-mercaptoethanol for 5 min and then separated in 10% polyacrylamide gels by SDS-PAGE. The proteins in the gels were then transferred to nitrocellulose membranes using a constant electrical current 0.37A for 1 hr at 4°. The membranes were probed with the following primary antibodies: anti-phospho-p44/42 MAPK rabbit monoclonal (D13.14.4E, Cell Signaling Technology) at 1:1,000 dilution, Mpk1 (E-9): sc-133189 mouse monoclonal (Santa Cruz Biotechnology) at 1:1,000 dilution and Phosphoglycerate kinase (Pgk1p) mouse monoclonal (Molecular Probes, Invitrogen) at 1:1,000 dilution. The primary antibodies were diluted in Odyssey blocking buffer (LI-COR) and incubated at room temperature (RT) for 1 hr. The membranes were washed four times with 1XPBS/ 0.2% Tween20 for 5 min per wash. The secondary antibodies used were Goat anti-Rabbit IRDye 800CW (1:10,000) LI-COR (green) and Goat anti-Mouse IRDye 680LT (1:10,000) LI-COR (red). The membranes were incubated with secondary antibodies for 30 min at RT and washed as described before. 1X PBS was used to wash quickly the membranes before detecting the proteins with the Odyssey CLx Infrared Fluorescent Imaging System.

### Viability assays and growth curves

Viability assays were performed using 1 × 10^8^ cell/ml aliquots taken from cultures at OD_600_ between 0.5-0.8. The strains bearing null mutations for single genes encoding bait sensor proteins, their corresponding protein partners, or double-mutant strains containing both deletions were tested with wild type strain BY4741 as a control (Table S1). The null mutants *cof1Δ*, *fas1Δ*, *fba1Δ*, *mtr3Δ*, *pga3Δ*, *rps31Δ*, and *ypl238cΔ* were not tested because these haploid null mutants are nonviable. The strains *lnp1Δ*, *ssb2Δ*, and *tef1Δ* were not available for this study. For agar plate assays, 3 μl drops taken from 1/10 serial dilutions of the working culture (ranging from 10^8^ to 10^2^ cell/ml) were inoculated in triplicate or greater on CSM agar plates under the following conditions: normal conditions (30°), heat stress (37°), oxidative stress (1 mM H_2_O_2_), plasma membrane stress (0.75μg/ml Amphotericin B), and cell wall stress (150 μg/ml Calcofluor white) and (75 ng/ml Caspofungin). Plates were incubated at 30° or 37° for 2-3 days. Relative growth on agar plates was quantified by comparing the extent of growth displayed by the wild type strain across all dilutions with the extent of growth displayed by the mutant strains. A phenotype was classified as resistant (R) if the mutant grew at a serial dilution greater than the wild-type; sensitive (S) if it grew at a serial dilution less than the wild-type; and wild type (WT) if it grew at a serial dilution equal to the wild-type. Growth curves from broth cultures were also performed to validate observations made in agar cultures by inoculating 1 × 10^8^ cell/ml aliquots in CSM broth medium and taking OD_600_ measurements every 15 min for up to 24 hr.

### Affinity Purification coupled to Mass Spectrometry

Mid2-TAP and Wsc1-TAP strains were grown in 40 ml of YPD at 30° overnight. The cells were centrifuged at 3,000 rpm for 3 min and washed three times with cold deionized water. The pellets were resuspended with 1 ml of lysis buffer (20 mM Hepes-KOH pH 7.4, 50 mM KOAc, 2 mM Mg(Ac)_2_, 10% glycerol, 2 mM CaCl_2_, 1% Triton X-100 and 1X Protease Inhibitor Cocktail). An equal volume of acid-glass beads was added to the cell pellets and the cells were disrupted by vortexing at maximum speed for 5 min in a cold room. The lysates were cleared by centrifugation at 4,000 rpm for 10 min. Afterward, 50 μl of calmodulin beads were equilibrated with 1ml of lysis buffer without Triton X-100 and then centrifuged at 1,500 rpm for 4 min. The beads were mixed with 400 μl of supernatant. The tube was placed in a rotator at 4° overnight. After incubation, the beads were centrifuged at 1,500 rpm for 4 min at 4° and the supernatants were removed. The beads were then washed three times with 800 μl of wash buffer 1 (30 mM Hepes- KOH pH 7.9, 150 mM NaCl, 1 mM imidazole, 2 mM CaCl_2_, 0.1% Triton X-100 and 1x Protease Inhibitor Cocktail) and then two times with 800 μl of wash buffer 2 (30 mM Hepes- KOH pH 7.9, 150 mM NaCl, 1 mM imidazole, 2mM CaCl_2_). For trypsin digestion, the beads were washed two times with 400 μl of digestion buffer (50 mM ammonium bicarbonate, 1 mM CaCl_2_) and the beads were resuspended with 50 μl of the same buffer. 2 μl of 100 mM tris (2-carboxy ethyl) phosphine (TCEP) were added to the mixture and the reaction was incubated for 1 hr at RT. 2 μl of 500 mM iodo-acetamide were added and incubated 50 min at RT protected from light. The reaction was quenched with 10 mM DTT and 1 μl of 1 mg/ml trypsin was added and the digestion was incubated overnight at 37° with shaking. To stop the digestion 1% glacial acetic acid was used. The samples were cleaned and desalted using C18 Zip-Tip and the peptides were eluted using 0.1% formic acid, 70% acetonitrile solution. The peptides were then dried by evaporation and resuspended with 1% formic acid. Positive hits were considered if they contained more than two unique peptides with not less than 92% probability and produced 0-1 peptide in the negative control samples.

### Immunoprecipitation coupled to Mass Spectrometry

5ml cultures of strains containing Mid2-GFP, Wsc1-GFP and BY4741 were grown in YPD at 30° overnight. 25 ml cultures of YPD media were inoculated with these overnight cultures and grown to an OD_600_∼1.0. The remaining seed culture was used for inoculation of a 1 L culture and incubated overnight. The cultures were centrifuged at 3,500 rpm for 15 min at 4°. The pellets were resuspended with an equal volume of IPLB (20 mM Hepes KOH, pH 7.4, 150 mM KOAc, 2 mM Mg(Ac)_2_, 1 mM EGTA, 10% glycerol and 1X protease inhibitor cocktail (PIC). The cells were lysed using a freezer mill and liquid nitrogen. The powders were thawed in dH_2_O bath at RT and 1X PIC was added. The lysed cells were centrifuged for 10 min at 2,000 rpm, 4° and the supernatants were transferred to a fresh tube. For the X-linking reaction, 2 ml of supernatant was mixed with 1 mM dithiobis(succinimidyl propionate) (DSP) and incubated on ice for 30 min with gentle inversion every 10 min. After incubation, 100 mM of 1 M Tris-HCl pH 7.5 was added and incubated on ice for 10 min with gentle inversion every 5 min. 1% of digitonin was added to the reaction and incubated on ice for 30 min. The samples were then centrifuged at 4,000 rpm for 15 min at 4°. 50 μl of μMACS Anti-GFP Microbeads (Miltenyi Biotec) were added to 1.5 ml of supernatant and incubated in ice for 30 min. The MACS columns (Miltenyi Biotec) in the magnetic holder were equilibrated with 250 μl of IPLB + 1% digitonin + 1X PIC. The supernatants were added to the columns and for the wash step the columns were washed with 1 ml IPLB + 0.1% digitonin + 1X PIC two times and one time with only 1 ml IPLB. For the on-bead digestion, 25 μl of EB I (2 M Urea, 50 mM Tris-HCl, pH 7.5, 25 mM DTT and 5 μg/ml trypsin) was added and incubated for 30 min at RT. 100 μl of EB II (2 M Urea, 50 mM Tris-HCl, pH 7.5 and 5 mM chloroacetamide) was added to the column and the eluted fractions were collected. The digestion was incubated overnight at RT and 1 μl of 100% TFA was added to stop the reaction. The sample was cleaned and desalted as previously described, except that the peptides were eluted using 1% acetic acid, 65% acetonitrile solution. The peptides were dried by evaporation and then resuspended with 1% formic acid.

### Calmodulin Affinity purification coupled to western blot

TAP-interactor strains transformed with a Mid2-HA or Wsc1-HA prey clones were inoculated in 5 ml Ura-/2% glucose media and incubated at 30°, 225 rpm, and overnight. 30 ml of Ura-/2% sucrose media were inoculated with 1 ml overnight culture and incubated as above. The cultures were centrifuged at RT at 3,000 rpm for 3 min. The pellets were washed twice with dH_2_O and resuspended with 1X YP/2% galactose media. The cultures were incubated for 3 hr. After incubation, the cultures were centrifuged at 4° and resuspended with 1 ml of Calmodulin-IPLB (CaM-IPLB) (10 mM Hepes-KOH pH 8, 150 mM NaCl, 1 mM MgOAc, 1 mM imidazole, 2 mM CaCl_2_). The pellets were resuspended with 1 ml CaM-IPLB plus 1% Triton X-100 and 1X Protease Inhibitor Cocktail set I from Roche. The cells were disrupted with glass beads for 5 min, vortexing at maximum speed in a cold room. The lysates were then centrifuged at 13,000 rpm for 5 min, at 4° and transferred to clean tubes. 10 μl of 1x Protease Cocktail Inhibitor were added to each lysate. The calmodulin sepharose beads were washed (2x) with CaM-IPLB and centrifuged at 3,000 rpm, 5 min, 4°. 50 μl of pre-washed calmodulin beads were added to the supernatants and incubated for 2 hr, at 4° in a rotator. The beads were centrifuged and washed 5x with 500 μl of CaM-IPLB. 50 μl of calmodulin elution buffer (10 mM Hepes-KOH pH 8, 150 mM NaCl, 1 mM MgOAc, 1 mM imidazole, 25 mM EGTA pH 8.0) was added to the beads. The beads were incubated for 5 min, centrifuged as above and the supernatants were used for analysis. Alternatively, the beads were resuspended with 50 μl of CaM-IPLB and denatured with 4X loading dye for 10 min at 95° and the supernatants were used for analysis.

### Data Availability

All strains are available upon request to the corresponding author. Supplemental Table S1: Strains used in this study. Supplemental Table S2: Oligonucleotides used in this study. Supplemental Figure S1: Nub G/I test for integrated C-tagged Wsc1p and Mid2p. Supplemental Figure S2: Localization of Wsc1p and Mid2p. Supplemental Figure S3: Validation of MYTH-tagged Mid2p and Wsc1p expression by Western blot. . Supplemental Figure S4: WSC1 and MID2 - iMYTH interactome showing only interactors identified in the iMYTH screen at 30°. Supplemental Figure S5: Growth curve analysis of single and double mutants exposed at different concentrations of Hydrogen Peroxide. Supplemental Figure S6: Growth curve analysis of single and double mutants exposed at different concentrations of Caspofungin. Supplemental Figure S7: Western blot analysis showing the phosphorylation of Slt2p in single and double mutant strains treated with 1mM hydrogen peroxide (H_2_O_2_) for 1 hr at 27°. Supplemental Figure S8: Western blot analysis showing the phosphorylation of Slt2p in single and double mutant strains treated with 75ng/ml Caspofungin for 1 hr at 27°. Supplemental Figure S9: Network graph of the Wsc1p and Mid2p interactome identified by iMYTH screen at 37°. Supplemental Table S3: Percentages of positive interactors for Wsc1p and Mid2p identified by two independent iMYTH screens performed at 37°. Supplemental Figure S10: A representative drop dilution assay of sensor and interactor null mutants exposed to stress conditions. Supplemental material available at Figshare: https://doi.org/10.25387/g3.7653122.

## Results

### Construction and confirmation of Wsc1p and Mid2p MYTH baits expression

To test whether Wsc1p and Mid2p interact with other signaling proteins *in vivo* we performed the iMYTH assay. First, we generated four different bait constructs; two in the yeast double reporter L40 strain (Wsc1-L40 L3 and Mid2-L40 L3) and two in the triple reporter THY.AP4 strain (Wsc1-THY L2 and Mid2-THY L3). The baits were endogenously tagged with Cub-LexA-VP16 (L2 version) or Cub-(YFP)-LexA-VP16 (L3 version) at their C-terminus and under the control of their native promoter.

To ensure that positive iMYTH results were not due to self-activation or spurious reactivity, the bait constructs were transformed with negative (N_ub_G) and positive (N_ub_I) control prey plasmids. The N_ub_I/N_ub_G test assesses whether the Wsc1 and Mid2 baits are being expressed and whether there is self-activation in the absence of an interacting protein. The Wsc1 THY L2, Wsc1 L40 L3, Mid2 THY L3 and Mid2 L40 L3 baits interacted with Ost1-N_ub_I and Fur4-N_ub_-I but did not interact with Ost1-N_ub_G and Fur4-N_ub_G indicating that the baits were correctly expressed and are not self-activated (Figure S1). An alternate verification method of bait expression is the *in vivo* localization test. As illustrated in Figure S2, the expressed Wsc1 L40 L3 tagged bait is localized at the emerging bud, whereas the Mid2 L40 L3 or Mid2 THY L3 tagged baits are uniformly distributed around the plasma membrane of the mother cells as reported in Straede and Heinisch 2007; Wilk *et al.* 2010; Delley and Hall 1999 and Piao *et al.* 2007.

To assess bait expression using western blot analysis, we used an antibody against the VP16 transcriptional activation domain of the MYTH cassette (Snider *et al.* 2010). All Wsc1p and Mid2p tagged at the C-terminus showed a band that is consistent with the size previously reported for Mid2p-HA (∼200KDa) (Ketela *et al.* 1999) and Wsc1p-HA (∼140KDa) (Lodder *et al.* 1999) (Figure S3).

In summary, the N_ub_I/N_ub_G protein expression, *in vivo* protein localization, and western blot assays ensured that the Wsc1p and Mid2p baits did not self-activate in the absence of prey and confirmed the expression and correct localization of these bait proteins.

### Wsc1 and Mid2 proteins interact with a diverse set of protein partners

**Figure 1:**
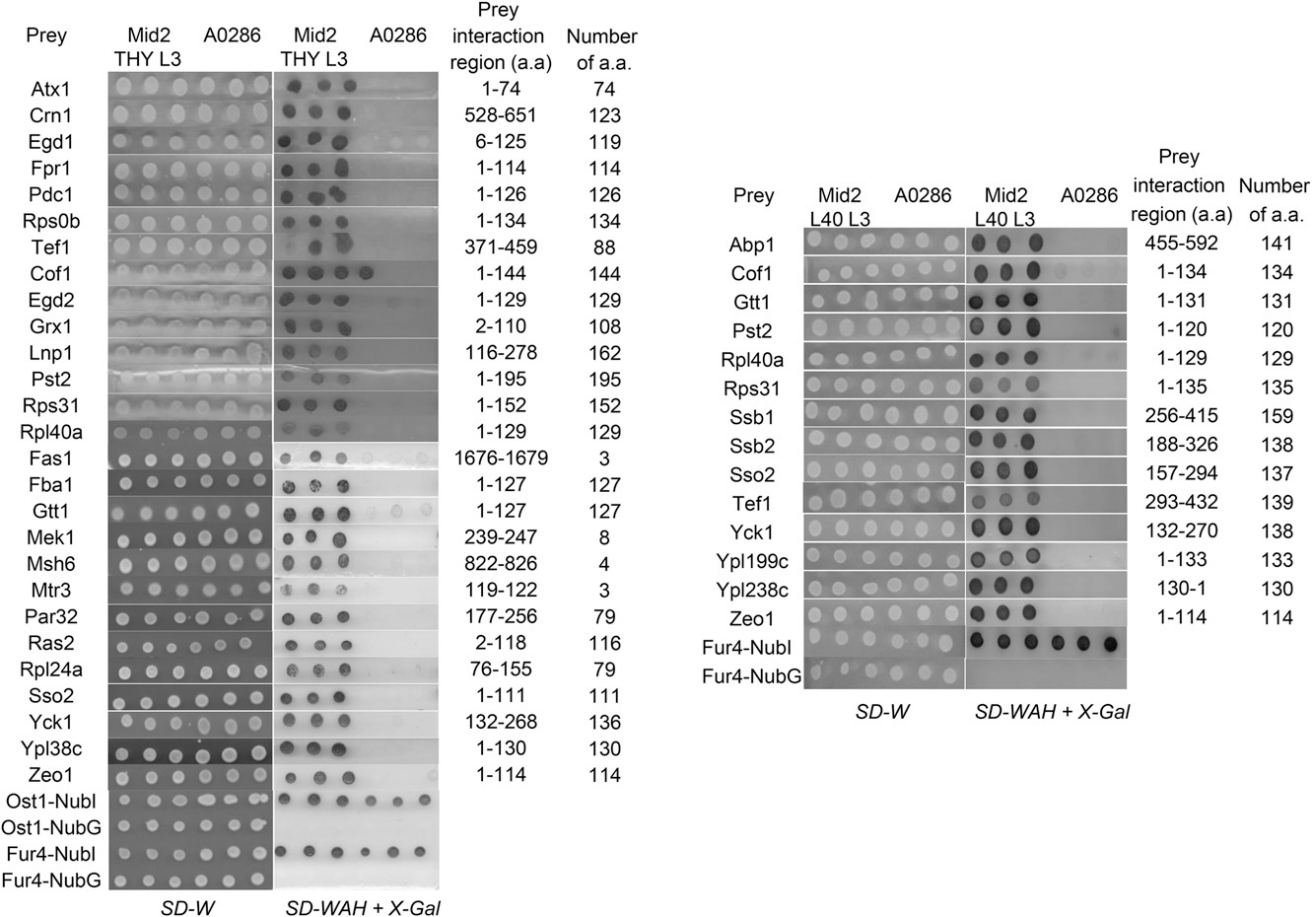
Yeast cells expressing Mid2p-Cub-TF were transformed with preliminary interactors and NubG/NubI control plasmids. Transformations were verified on *SD-W* medium. Interaction between the bait and prey was indicated by growth on *SD-WAH or SD-WH + X-Gal*. Transformants were spotted in triplicates. (Left panel: THYAP4 background and on right panel: L40 background).

**Figure 2:**
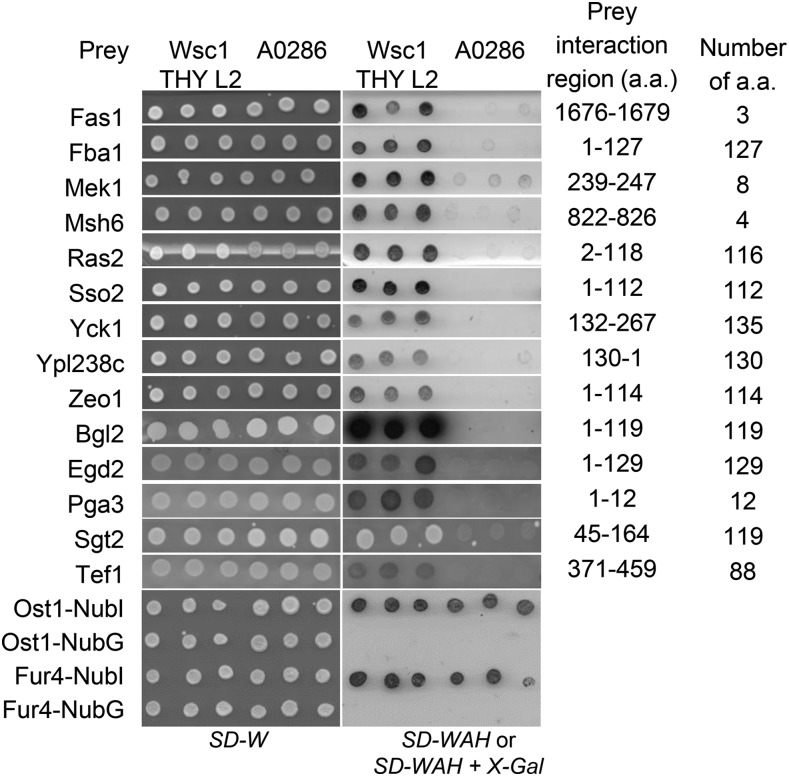
Yeast cells expressing Wsc1p-Cub-TF were transformed with preliminary interactors and NubG/NubI control plasmids. Transformations were verified on *SD-W* medium. Interaction between the bait and prey was indicated by growth on *SD-WAH or SD-WAH + X-Gal*. Transformants were spotted in triplicates.

Wsc1p and Mid2p bait strains were transformed with a pNubG-X cDNA library (Dualsystems). Preys that interacted with the sensor baits were selected from interaction media and the plasmids were recovered and sequenced. These plasmids were also used to transform Wsc1 and Mid2 bait strains a second time for validation of the interactions in bait dependency tests (Snider *et al.* 2010). To compare the specificity of interactors with our bait protein, an artificial bait (A0286 or A0287) acting as a negative control which has a very ‘minimal’ coding sequence consisting of only the Cub-LexA-VP16 tag and a short sequence necessary to direct it to the plasma membrane was used. Also, to determine if an interactor is unique for the bait with which it was identified, the Mid2-interacting proteins were transformed into a Wsc1p bait strain and vice versa (data not shown). Overall, out of 552 initial positive colonies selected for Wsc1p, and ∼660 initial positive colonies for Mid2p, the bait dependency tests confirmed a total of 34 protein interactors; 31 interactors for Mid2p and 14 interactors for Wsc1p of which 11 were shared between them. All interactors were bait-specific as they did not interact with A0286 or A0287 negative controls (Figures 1 and 2 respectively).

**Figure 3:**
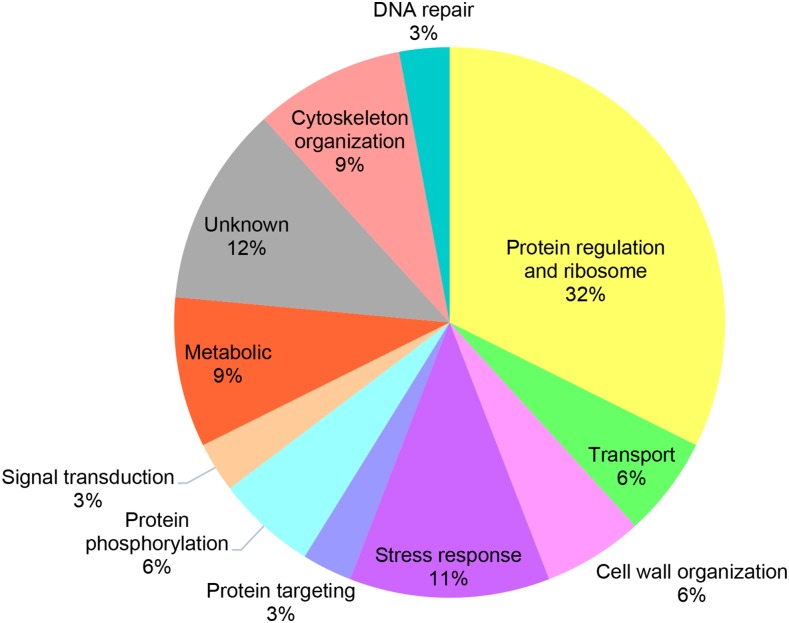
The 34 iMYTH interactors of Wsc1p and Mid2p have been classified by their corresponding gene ontology annotations according to eleven biological processes. Each of the percentages listed reflect the number of protein interactors in each biological process category from a total of 34.

The “Wsc1” and “Mid2” interactomes identified by iMYTH are listed in Table 1 and their relative representation and biological functions are specified in Figure 3. From these results we conclude that under normal vegetative growth conditions, Wsc1p and Mid2p interact with a diverse set of cytoplasmic and plasma membrane proteins of which 11 are shared between both sensors and the remaining proteins are novel.

**Table 1:** Thirty seven Wsc1and Mid2-interacting proteins identified by iMYTH at 30° and 37°

Gene name	Systemic name	Bait	Description (According to SGD, http://www.yeastgenome.org/)	Null mutation viability
ABP1	YCR088W	MID2	Actin-binding protein of the cortical actin cytoskeleton; important for activation of actin nucleation mediated by the Arp2/Arp3 complex; inhibits actin filament elongation at the barbed-end; phosphorylation within its proline-rich region, mediated by Cdc28p and Pho85p, protects Abp1p from PEST sequence-mediated proteolysis; mammalian homolog of HIP-55 (hematopoietic progenitor kinase 1 [HPK1]-interacting protein of 55 kD)	viable
ADE2 (37°)	YOR128C	WSC1	Phosphoribosylaminoimidazole carboxylase; catalyzes a step in the ’*de novo*’ purine nucleotide biosynthetic pathway; red pigment accumulates in mutant cells deprived of adenine	viable
ATX1	YNL259C	MID2	Cytosolic copper metallochaperone; transports copper to the secretory vesicle copper transporter Ccc2p for eventual insertion into Fet3p, which is a multicopper oxidase required for high-affinity iron uptake; human homolog ATOX1 can complement yeast atx1 mutant; overexpression of human ATOX1 suppresses lysine auxotrophy of the yeast sod1 null mutant, as does overexpression of yeast ATX1	viable
BGL2	YGR282C	WSC1	Endo-beta-1,3-glucanase; major protein of the cell wall, involved in cell wall maintenance; involved in incorporation of newly synthesized mannoprotein molecules into the cell wall	viable
COF1	YLL050C	MID2	Cofilin, involved in pH-dependent actin filament depolarization; binds both actin monomers and filaments and severs filaments; involved in the selective sorting, export of the secretory cargo from the late golgi; genetically interacts with pmr1; thought to be regulated by phosphorylation at SER4; ubiquitous and essential in eukaryotes	nonviable
CRN1	YLR429W	MID2	Coronin, cortical actin cytoskeletal component that associates with the Arp2p/Arp3p complex to regulate its activity; plays a role in regulation of actin patch assembly	viable
EGD1	YPL037C	MID2	Subunit beta1 of the nascent polypeptide-associated complex (NAC); involved in protein targeting, associated with cytoplasmic ribosomes; enhances DNA binding of the Gal4p activator; homolog of human BTF3b; EGD1 has a paralog, BTT1, that arose from the whole genome duplication	viable
EGD2 (30°/37°)	YHR193C	WSC1/MID2	Alpha subunit of the nascent polypeptide-associated complex (NAC); involved in protein sorting and translocation; associated with cytoplasmic ribosomes	viable
FAS1	YKL182W	WSC1/MID2	Beta subunit of fatty acid synthetase; complex catalyzes the synthesis of long-chain saturated fatty acids; contains acetyltransacylase, dehydratase, enoyl reductase, malonyl transacylase, and palmitoyl transacylase activities	nonviable
FBA1	YKL060C	WSC1/MID2	Fructose 1,6-bisphosphate aldolase; required for glycolysis and gluconeogenesis; catalyzes conversion of fructose 1,6 bisphosphate to glyceraldehyde-3-P and dihydroxyacetone-P; locates to mitochondrial outer surface upon oxidative stress; N-terminally propionylated *in vivo*	nonviable
FPR1	YNL135C	MID2	Peptidyl-prolyl *cis*-trans isomerase (PPIase), binds to the drugs FK506 and rapamycin; also binds to the nonhistone chromatin binding protein Hmo1p and may regulate its assembly or function; N-terminally propionylated *in vivo*; mutation is functionally complemented by human FKBP1A	viable
GRX1	YCL035C	MID2	Glutathione-dependent disulfide oxidoreductase; hydroperoxide and superoxide-radical responsive, heat-stable, with active site cysteine pair; protects cells from oxidative damage; GRX1 has a paralog, GRX2, that arose from the whole genome duplication; protein abundance increases in response to DNA replication stress	viable
GTT1	YIR038C	MID2	ER associated glutathione S-transferase; capable of homodimerization; glutathione transferase for Yvc1p vacuolar cation channel; expression induced during the diauxic shift and throughout stationary phase; functional overlap with Gtt2p, Grx1p, and Grx2p	viable
LNP1	YHR192W	MID2	Lunapark family member involved in ER network formation; regulates the ER asymmetry-induced inheritance block during ER stress; localizes to ER junctions and this localization is regulated by the yeast atlastin ortholog Sey1p; interacts with the reticulon protein Rtn1p; induced in response to the DNA-damaging agent MMS	viable
MEK1	YOR351C	WSC1/MID2	Meiosis-specific serine/threonine protein kinase; functions in meiotic checkpoint, promotes recombination between homologous chromosomes by suppressing double strand break repair between sister chromatids; stabilizes Hop1-Thr318 phosphorylation to promote interhomolog recombination and checkpoint responses during meiosis	viable
MSH6	YDR097C	WSC1/MID2	Protein required for mismatch repair in mitosis and meiosis; forms a complex with Msh2p to repair both single-base & insertion-deletion mispairs; also involved in interstrand cross-link repair; potentially phosphorylated by Cdc28p	viable
MTR3	YGR158C	MID2	Exosome non-catalytic core component; involved in 3′-5′ RNA processing and degradation in both the nucleus and the cytoplasm; has similarity to *E. coli* RNase PH and to human hMtr3p (EXOSC6)	nonviable
PAR32	YDL173W	MID2	Protein of unknown function; hyperphosphorylated upon rapamycin treatment in a Tap42p-dependent manner; green fluorescent protein (GFP)-fusion protein localizes to the cytoplasm; PAR32 is not an essential gene	viable
PDC1	YLR044C	MID2	Major of three pyruvate decarboxylase isozymes; key enzyme in alcoholic fermentation; decarboxylates pyruvate to acetaldehyde; subject to glucose-, ethanol-, and autoregulation; involved in amino acid catabolism; activated by phosphorylation in response to glucose levels; N-terminally propionylated *in vivo*	viable
PGA3	YML125C	WSC1	Putative cytochrome b5 reductase, localized to the plasma membrane; may be involved in regulation of lifespan; required for maturation of Gas1p and Pho8p, proposed to be involved in protein trafficking	nonviable
PST2	YDR032C	MID2	Protein with similarity to a family of flavodoxin-like proteins; induced by oxidative stress in a Yap1p dependent manner; the authentic, non-tagged protein is detected in highly purified mitochondria in high-throughput studies; protein abundance increases in response to DNA replication stress; PST2 has a paralog, RFS1, that arose from the whole genome duplication	viable
RAS2 (30°/37°)	YNL098C	WSC1/MID2	GTP-binding protein; regulates nitrogen starvation response, sporulation, and filamentous growth; farnesylation and palmitoylation required for activity and localization to plasma membrane; homolog of mammalian Ras proto-oncogenes; RAS2 has a paralog, RAS1, that arose from the whole genome duplication	viable
RPL11B (37°)	YGR085C	MID2	Ribosomal 60S subunit protein L11B; expressed at half the level of Rpl11Ap; involved in ribosomal assembly; depletion causes degradation of 60S proteins and RNA; homologous to mammalian ribosomal protein L11 and bacterial L5; RPL11B has a paralog, RPL11A, that arose from the whole genome duplication	viable
RPL24A	YGL031C	MID2	Ribosomal 60S subunit protein L24A; not essential for translation but may be required for normal translation rate; homologous to mammalian ribosomal protein L24, no bacterial homolog; RPL24A has a paralog, RPL24B, that arose from the whole genome duplication	viable
RPL40A	YIL148W	MID2	Ubiquitin-ribosomal 60S subunit protein L40A fusion protein; cleaved to yield ubiquitin and ribosomal protein L40A; ubiquitin may facilitate assembly of the ribosomal protein into ribosomes; homologous to mammalian ribosomal protein L40, no bacterial homolog; RPL40A has a paralog, RPL40B, that arose from the whole genome duplication; relative distribution to the nucleus increases upon DNA replication stress	viable
RPS0B	YLR048W	MID2	Protein component of the small (40S) ribosomal subunit; RPS0B has a paralog, RPS0A, that arose from the whole genome duplication; required for maturation of 18S rRNA along with Rps0Ap; deletion of either RPS0 gene reduces growth rate, deletion of both genes is lethal; homologous to human ribosomal protein SA and bacterial S2	viable
RPS1B (37°)	YML063W	MID2	Ribosomal protein 10 (rp10) of the small (40S) subunit; homologous to mammalian ribosomal protein S3A, no bacterial homolog; RPS1B has a paralog, RPS1A, that arose from the whole genome duplication	viable
RPS31	YLR167W	MID2	Fusion protein cleaved to yield ribosomal protein S31 and ubiquitin; ubiquitin may facilitate assembly of the ribosomal protein into ribosomes; interacts genetically with translation factor eIF2B	nonviable
SGT2	YOR007C	WSC1	Glutamine-rich cytoplasmic cochaperone; serves as a scaffold bringing together Get4, Get5p, and other TRC complex members that are required to mediate posttranslational insertion of tail-anchored proteins into the ER membrane; interacts with the prion domain of Sup35p; amyloid sensor; plays a role in targeting chaperones to prion aggregates; similar to human cochaperone SGT; forms cytoplasmic foci upon DNA replication stress	viable
SSB1	YDL229W	MID2	Cytoplasmic ATPase that is a ribosome-associated molecular chaperone, functions with J-protein partner Zuo1p; may be involved in folding of newly-made polypeptide chains; member of the HSP70 family; interacts with phosphatase subunit Reg1p; SSB1 has a paralog, SSB2, that arose from the whole genome duplication	viable
SSB2	YNL209W	MID2	Cytoplasmic ATPase that is a ribosome-associated molecular chaperone, functions with J-protein partner Zuo1p; may be involved in the folding of newly-synthesized polypeptide chains; member of the HSP70 family; SSB2 has a paralog, SSB1, that arose from the whole genome duplication	viable
SSO2	YMR183C	WSC1/MID2	Plasma membrane t-SNARE; involved in fusion of secretory vesicles at the plasma membrane; syntaxin homolog that is functionally redundant with Sso1p; SSO2 has a paralog, SSO1, that arose from the whole genome duplication	viable
TEF1	YPR080W	WSC1/MID2	Translational elongation factor EF-1 alpha; GTP-bound active form, binds to and delivers aminoacylated tRNA to the A-site of ribosomes for elongation of nascent polypeptides; moonlighting function as an actin binding and bundling protein; association with GTPase Rho1p on the vacuolar membrane may facilitate F-actin remodeling; involved in tRNA re-export from the nucleus	viable
YCK1	YHR135C	WSC1/MID2	Palmitoylated plasma membrane-bound casein kinase I (CK1) isoform; shares redundant functions with Yck2p in morphogenesis, proper septin assembly, endocytic trafficking, and glucose sensing; stabilized by Sod1p binding in the presence of glucose and oxygen, causing glucose repression of respiratory metabolism; involved in the phosphorylation and regulation of glucose sensor Rgt2p; YCK1 has a paralog, YCK2, that arose from the whole genome duplication	viable
YPL199C	YPL199C	MID2	Putative protein of unknown function, predicted to be palmitoylated	viable
YPL238C	YPL238C	WSC1/MID2	Dubious open reading frame; unlikely to encode a functional protein, based on available experimental and comparative sequence data; partially overlaps 5′ end of the verified essential gene SUI3/YPL237W	nonviable
ZEO1 (30°/37°)	YOL109W	WSC1/MID2	Peripheral membrane protein of the plasma membrane; interacts with Mid2p; regulates the cell integrity pathway mediated by Pkc1p and Slt2p; the authentic protein is detected in a phosphorylated state in highly purified mitochondria	viable

### Validation of iMYTH interactors by alternative biochemical methods

**Figure 4:**
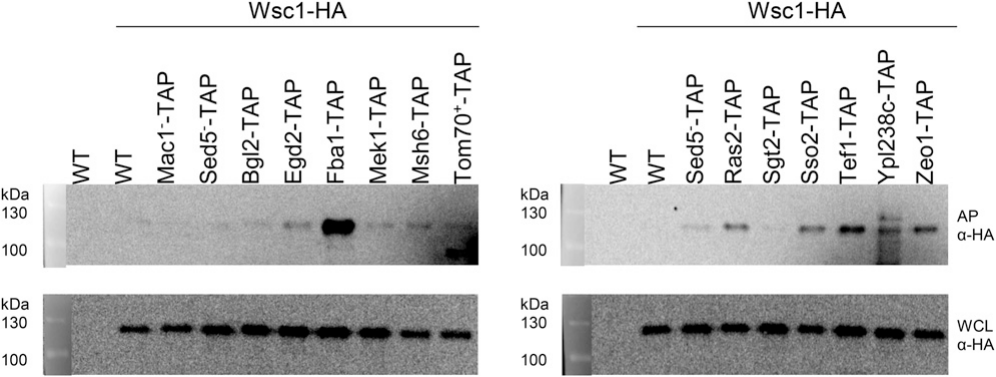
Affinity purification of TAP-tagged bait proteins using calmodulin-sepharose beads. In this experimental design, the interactor proteins were used as TAP-tagged baits to capture Wsc1-HA preys. The strains were first incubated in medium without Uracil selection to retain the prey plasmid and were subsequently induced by YP/galactose medium to express the prey proteins. Expression of Wsc1-HA preys was verified in whole cell lysates (WCL). Mac1p and Sed5p are negative interaction controls. Tom70p is a positive interaction control. WT and WT’ negative controls were described in the text. Mek1p and Msh6p, although confirmed in this assay, were not included subsequently in the interactome in Figure 5 because these interactors were represented by open reading frames of 8 and 4 a.a. respectively and were considered too short for further consideration (See Figures 1 and 2).

To further validate the iMYTH interactome results, we performed complementary assays using calmodulin affinity purification coupled to mass spectrometry (AP-MS), immunoprecipitation coupled to mass spectrometry (IP-MS), or calmodulin affinity purification coupled to western blotting (AP-WB). To simplify our AP-WB assay, we reversed the protein capture scheme by using the iMYTH prey proteins as TAP-tagged baits (Ghaemmaghami *et al.* 2003) and expressing Mid2-HA or Wsc1-HA as prey proteins. The TAP-tagged baits were purified with calmodulin beads. In this way, the interactions between Wsc1-HA and the interactors Egd2p, Fba1p, Mek1p, Msh6p, Ras2p, Sso2p, Tef1p, Ypl238cp, and Zeo1p were validated by AP-WB (Figure 4, top two panels). The WT negative control in lane 1 (top two panels) expressed no recombinant Wsc1-HA while the second WT’ negative control in lane 2 (top two panels) expressed Wsc1-HA but did not express a bait protein. Both negative controls generated no bands in the top panel indicating that all prey protein interactions were specific for each of the bait proteins assayed. Negative bait controls Mac1-TAP and Sed5-TAP were selected because these proteins are not reported to interact with Wsc1p. These did not interact with Wsc1p in this AP-WB assay. Positive control Tom70-TAP generated a faint positive band. Tom70p was previously described as physical interactors of Wsc1p using the PCA method (Miller 2005). The strong signal exhibited by Fba1p is probably due to the relatively high abundance of this protein in the cell (∼8.5x10^5^ molecules/cell, (Kulak *et al.* 2014)) and is considered a positive interactor. Altogether, these control experiments support that the positive iMYTH protein bands represent a subset of protein interactions validated by AP-WB. Mid2-HA bands captured by the same AP-WB assay were difficult to focus because of their extensive glycosylation and could not be validated.

For AP-MS assays, we used endogenously Wsc1-TAP and Mid2-TAP tagged baits and purified the complexes using the approach described in the Materials and Methods section. As negative controls, total protein extracts obtained from WT strains expressing no recombinant baits were used. Affinity purifications using whole cell extracts from WT negative control samples generated no peptides thus validating the experimental results obtained with the Wsc1p and Mid2p baits (Table 2). Rpl40a protein was the only interaction confirmed for Mid2p using this method.

**Table 2:** Exclusive unique peptide count for a new Mid2p interactor identified by iMYTH and validated by calmodulin affinity purification-mass spectrometry analysis (AP-MS)

Protein name	Wt			Wsc1			Mid2		
	TP	TP	TP	TP	TP	TP	TP	TP	TP
Wsc1	0	0	0	3(92.95)	0	3(99.09)	0	0	0
Mid2	0	0	0	0	0	0	19 (99.58)	18 (99.58)	4 (99.58)
Rpl40a	0	0	0	2(99.58)	0	0	3 (99.58)	2 (99.58)	2 (99.58)

In parenthesis the probability percentage, N= 3.

**Figure 5:**
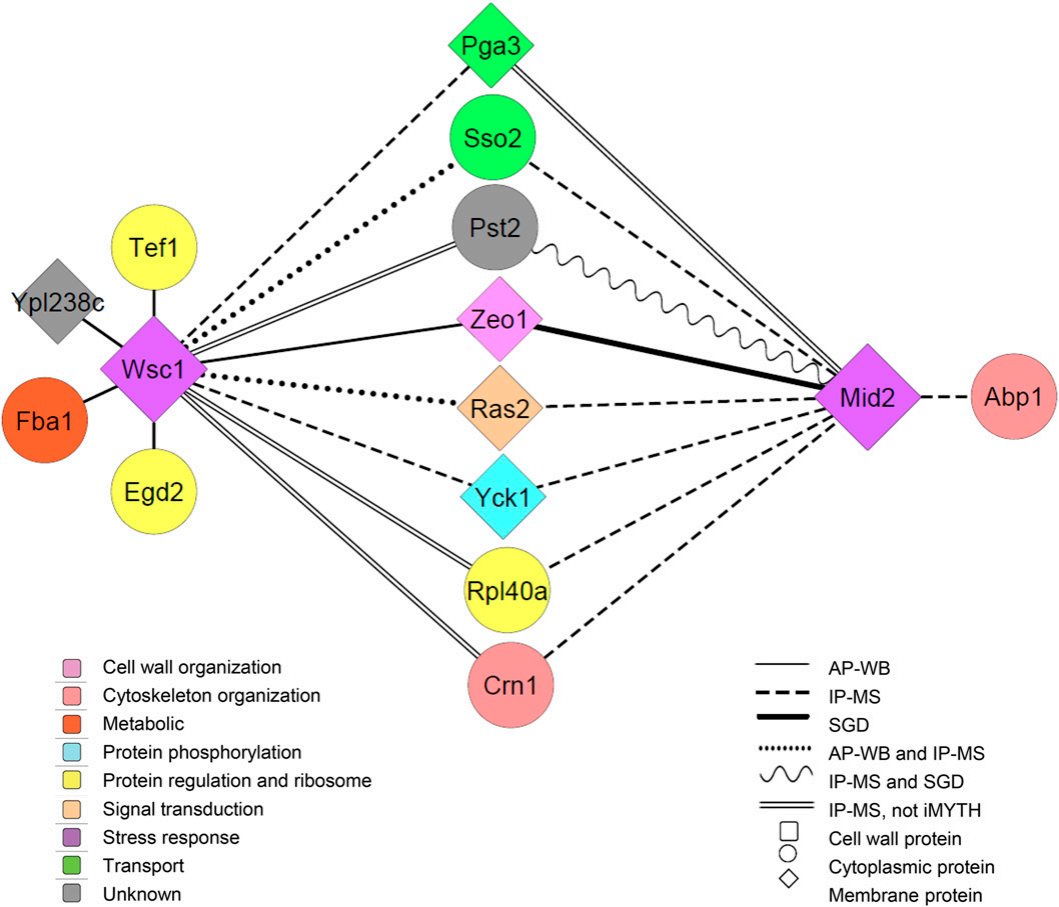
Proposed physical interactome for Wsc1p and Mid2p as determined by iMYTH with validation by at least one alternative physical method. Zeo1p and Pst2p are protein interactors that were previously reported in the SGD as interactors for Mid2p. For this reason these were connected to Mid2p in this interactome. Color codes indicate biological and biochemical functions.

We also performed the IP-MS method as an alternative assay to confirm Wsc1p and Mid2p interactions. Samples that generated 0-1 peptides in the wild type (WT) negative control sample and two or more peptides with > 92% probability in experimental samples were considered valid (Tables 3 and 4). Up to two peptides were tolerated in the WT negative control sample when double-digit numbers of peptides were obtained in experimental samples (for example Crn1 in Table 3). The IP-MS assay confirmed 3 interactors of Wsc1p and 7 of Mid2p. Interactors Pst2p, Crn1p, Rpl40ap and Pga3p also interacted with both Wsc1p and Mid2p. Based on secondary validation results of the iMYTH protein-protein interactions by alternative physical methods, we propose a second less complex interactome representing the most stable interactors for Wsc1p and Mid2p (Figure 5). This interactome consists of 8 shared interactors of Wsc1p and Mid2p, six unique interactors of Wsc1p, and one unique interactor for Mid2p. For completeness, all iMYTH interactors of Wsc1p and Mid2p that were previously annotated in the SGD are also included irrespective of their validation status.

**Table 3:** Exclusive unique peptide count for a new Wsc1p and Mid2p interactor identified by iMYTH and validated by immunoprecipitation coupled to mass spectrometry analysis (IP-MS)

Protein name	Wt			Wsc1			Mid2		
	TP	TP	TP	TP	TP	TP	TP	TP	TP
Wsc1	0	0	0	0	0	0	0	0	0
Mid2	0	0	0	0	0	0	0	0	0
Yck1	0	0	0	5(99.58)	3(99.58)	5(99.58)	3(99.58)	3(99.58)	2(99.58)
Rpl40a	0	0	0	5(99.58)	5(99.58)	5(99.58)	7(99.58)	26(99.58)	2 (99.58)
Crn1	0	2(99.29)	0	96(99.58)	105(99.5)	93(99.58)	41(99.58)	41(99.58)	40(99.58)
Pga3	0	0	0	7(99.58)	4(99.58)	9(99.58)	5(99.58)	3(99.58)	5(99.58)
Sso2	0	0	1(90.87)	2(96.26)	3(99.58)	2(97.16)	2(95.66)	2(96.63)	2(94.64)

In parenthesis the probability percentage, N= 3.

**Table 4:** Exclusive unique peptide count for new Wsc1p and Mid2p interactors identified by iMYTH and validated by immunoprecipitation coupled to mass spectrometry analysis (IP-MS)

Protein name	Wt		Wsc1		Mid2	
	TP	%	TP	%	TP	%
Wsc1	0	—	2	99.58	0	—
Mid2	0	—	0	—	19	99.58
Abp1	0	—	0	—	5	99.58
Pst2	0	—	25	99.58	15	99.58
Ras2	0	—	17	99.58	25	99.58
Rpl40a	0	—	9	99.58	8	99.58
Sso2	0	—	4	99.52	5	99.58
Yck1	0	—	3	99.58	5	99.58

TP= Total peptide %= Probability N = 1.

### Functional testing identifies stress-specific interactions

To test the biological importance of the protein interactions identified for Wsc1p and Mid2p, we tested the requirement of these protein interactors to maintain yeast cell fitness under stress conditions induced by Hydrogen peroxide (H_2_O_2_), Calcofluor white (CFW), Caspofungin (CSP) and Amphotericin b (AMPB), four chemical agents known for their antifungal properties.

The tested strains contained a single deletion of the interactor protein gene, or its corresponding stress sensor gene, or a double deletion containing both the stress sensor and each interactor gene. The relative growth of each test strain culture under no-stress or stress conditions was compared to the growth of a wild-type control strain. The aggregated results of three or more experiments are shown (Table 5 and Figure S10).

**Table 5:** Susceptibility of deletion strains to antifungal treatments*

Strain	1mM H_2_O_2_	150 µg/ml CFW	75 ng/ml CSP	0.75 μg/ml AMPB
*wsc1∆*	S	S	S	Wt
*mid2∆*	S	Wt	S	Wt
*abp1Δ*	Wt	Wt	Wt	Wt
*atx1Δ*	S	Wt	S	Wt
*bgl2Δ*	Wt	Wt	Wt	Wt
*crn1Δ*	Wt	Wt	Wt	Wt
*egd1Δ*	Wt	Wt	Wt	Wt
*egd2Δ*	Wt	Wt	Wt	Wt
*fpr1Δ*	Wt	Wt	Wt	Wt
*grx1Δ*	S	Wt	S	Wt
*gtt1Δ*	Wt	Wt	Wt	Wt
*msh6Δ*	Wt	Wt	Wt	Wt
*mek1∆*	Wt	Wt	Wt	Wt
*par32Δ*	Wt	Wt	Wt	Wt
*pdc1Δ*	Wt	Wt	Wt	Wt
*pst2Δ*	Wt	Wt	Wt	Wt
*ras2Δ*	S	Wt	Wt	Wt
*rpl24aΔ*	Wt	Wt	Wt	Wt
*rpl40aΔ*	Wt	Wt	S	Wt
*rps0bΔ*	Wt	Wt	Wt	Wt
*sgt2Δ*	Wt	Wt	Wt	Wt
*ssb1Δ*	Wt	Wt	S	Wt
*sso2Δ*	Wt	Wt	Wt	Wt
*yck1Δ*	Wt	Wt	Wt	Wt
*ypl199cΔ*	Wt	Wt	Wt	Wt
*zeo1Δ*	Wt	Wt	Wt	Wt
*atx1Δmid2Δ*	S	—	S	—
*grx1Δmid2Δ*	S	—	S	—
*ras2Δmid2Δ*	S	—	S	—
*ras2Δwsc1Δ*	S	—	S	—
*zeo1Δmid2Δ*	—	—	Wt	—
*zeo1Δwsc1Δ*	—	—	S	—

*cof1Δ*, *fas1Δ*, *fba1Δ*, *mtr3Δ*, *pga3Δ*, *rps31Δ*, and *ypl238cΔ* strains were not tested because are nonviable. The strains *lnp1Δ*, *ssb2Δ*, and *tef1Δ* were not available in this study. H_2_O_2_ = Hydrogen Peroxide; CFW = Calcofluor White; CSP = Caspofungin; AMPB = Amphotericin B; Wt = growth equivalent to wild type; S = sensitive or growth two dilutions less than wild type; - = not tested.

* A representative drop dilution assay is shown in Figure S10.

As expected from previous reports, the *wsc1∆* strain was sensitive (S) to CFW (Leduc *et al.* 2003), yet none of the Wsc1p interactors were sensitive to CFW and exhibited a wild-type growth phenotype (Wt). A *mid2∆* mutant was resistant in CFW as was previously reported (Ketela *et al.* 1999). The *wsc1∆* strain exhibited the greatest sensitivity to the treatments that were applied (3 of the 4 treatments tested), *mid2∆*, *atx1∆*, and *grx1∆* each exhibited sensitivity to 2 of the 4 treatments tested, and *ras2∆*, *rpl40a∆*, and *ssb1∆* mutants were sensitive to only 1 of the 4 of the treatments tested. The remaining deletion strains exhibited resistance similar to the wild type strain.

**Figure 6:**
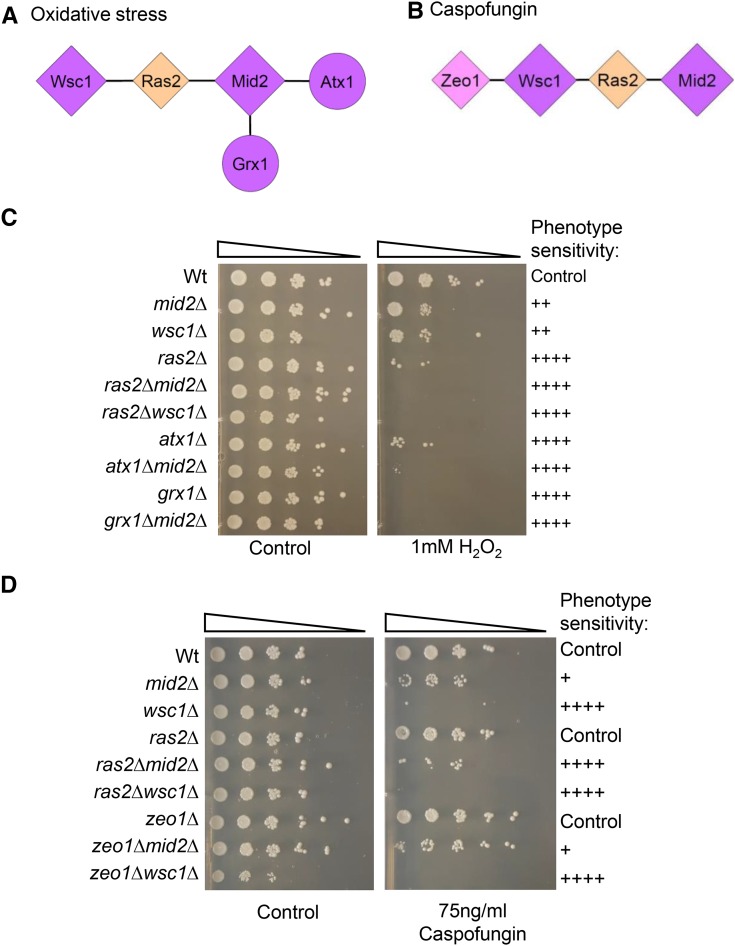
Components of putative signaling complex required for resistance to oxidative stress (A) and Caspofungin (B). Hydrogen Peroxide and Caspofungin interactomes refined from the functional test results. The Caspofungin interactome included Ras2 and Zeo1 signaling proteins. Viability analysis of single and double mutants exposed at different concentrations of Hydrogen Peroxide (C) and Caspofungin (D). The plates were inspected after 3 days of incubation. Color codes for A and B are as in Figure 5.

To identify specific bait/prey interactions that may be meaningful for survival upon exposure to a particular antimicrobial agent, we focused our attention to those protein interactors that if deleted, exhibited a growth phenotype similar to a deletion of their cognate sensor protein gene or enhanced sensitivity in a double deletion combination with their sensor gene, under the same treatment regimen (Table 5). By these criteria, proteins Wsc1, Mid2, Ras2, Atx1, Grx1 and Zeo1 were selected (Table 5). These sensitivity phenotypes lead us to propose two putative signaling complexes involved in the stress response to oxidative stress with 1 mM Hydrogen Peroxide (Figure 6A) and cell wall stress with 75 ng/ml Caspofungin (Figure 6B) treatments.

In serial dilution culture assays, the *wsc1*∆ and *mid2*∆ strains both shared enhanced sensitivity to 1 mM H_2_O_2_ treatment (Figure 6C) but not at lower concentrations when compared to wild type control strains (data not shown). In this assay, Wsc1p exhibited a similar growth phenotype under oxidative stress by hydrogen peroxide as Mid2p. Deletion of Mid2p interactors *ras2∆*, *atx1∆*, and *grx1∆* caused enhanced sensitivity to H_2_O_2_ at concentrations of 1 mM H_2_O_2_ (Figure 6C). Similar results were observed in broth cultures (Figure S5). The *ras2∆wsc1∆ d*ouble mutant strain as did *ras2∆mid2∆* exhibited a synthetic growth defect under oxidative stress conditions with 1 mM H_2_O_2_ whereas the remaining Wsc1p interactors’ single mutants were resistant (Wt) (Table 5) and were not tested further. These results suggested that the interacting protein pairs Ras2p-Wsc1p and Ras2p-Mid2p are required for resistance to oxidative stress conditions (Figure 6C).

**Figure 7:**
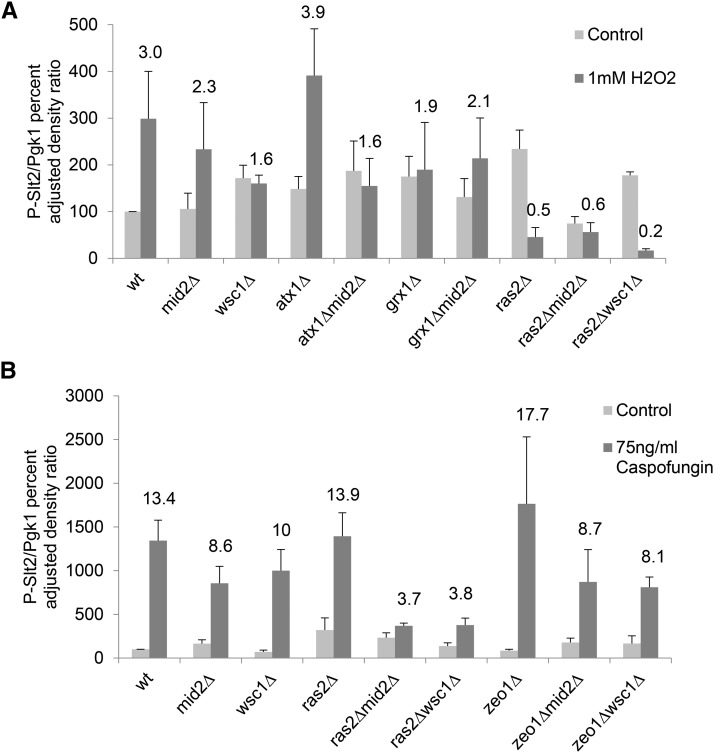
Western blot densitometry analysis of phospho-Slt2p levels in null mutants treated with 1mM H_2_O_2_ (A) and 75ng/mL of Caspofungin (B). Wild type, single and double mutants cultures at OD_600_ ∼0.7-0.9 were incubated with or without the treatment for 1 hr at 27°. Extracts prepared from each strain were immunoblotted with anti-phospho p44/42 MAPK rabbit monoclonal antibody (or P-Slt2p). The intensities of P-Slt2p were measured and normalized to Pgk1p level. The values are plotted as the fold change respect to wild type cells at 27° (Control). The number above the dark gray bars represent the fold change relative to the wild type control. A representative blot image of H_2_O_2_ and Caspofungin is showed in the Figure S7 and S8, respectively. The data shown mean ± SEM of n ≥ 3.

The *wsc1∆* and *mid2∆* strains both shared increased sensitivity to 75 ng/ml Caspofungin treatment (Figure 6D). Similar results were observed when we compared their growth phenotypes with growth curves in broth cultures (Figure S6). Though the *ras2∆* and *zeo1∆* mutants did not exhibit any growth sensitivity to Caspofungin compared to the wild-type (Figure 6D), they exhibited a strong phenotype when coupled with their corresponding sensor mutant in the double mutant combinations: *ras2∆mid2∆*, *ras2∆wsc1∆*, *zeo1∆mid2∆*, and *zeo1∆wsc1∆*, and were thus included in the proposed Caspofungin signaling complex (Figure 6B). The *zeo1∆mid2∆* mutant combination showed the weakest genetic interaction effect of the four tested. These double mutant strains were each tested for phosphorylation status of Slt2p (Figure 7).

### The Ras2p, Grx1p and Zeo1p are not required for Slt2p phosphorylation in response to Caspofungin treatment while Ras2p is required for Slt2p phosphorylation in response to oxidative stress

To assess if the observed growth reduction in null single and double mutant strains could be associated with Slt2p regulation, we conducted western blot analysis of Slt2p/Mpk1p phosphorylation (P-Slt2p) status in strains cultured in the presence of 1 mM H_2_O_2_ or 75 ng/ml Caspofungin (Figures 7A and 7B respectively).

Exposure of yeast cultures to 1 mM H_2_O_2_ induces accumulation of P-Slt2p in the wild type control strain (average of triplicate experiments shown in Figure 7A, and an illustrative example shown in Figure S7). In the *mid2∆* and *atx1∆* single mutants, there was accumulation of P-Slt2p in the presence of 1 mM H_2_O_2_ similar to the wild type control strain. Thus, the phosphorylation status of Slt2p could not be correlated with the growth inhibition phenotype in these strains. However, the *mid2∆atx1∆* double mutant combination exhibited a genetic interaction that prevented the accumulation of P-Slt2p in response to oxidative stress (Figure 7A). The *grx1∆* single mutant and *mid2∆grx1∆* double mutant strains were each unable able to accumulate the P-Slt2p. Thus, in the case of the interaction between Grx1p and Mid2p, the phosphorylation status of Slt2p and the growth inhibition phenotype were correlated.

Deletion of the *RAS2* gene increased baseline phosphorylation of Slt2p in the absence of 1 mM H_2_O_2_ treatment (Figure 7A). However, when treated with 1mM H_2_O_2_ the *ras2*∆ mutant failed to accumulate P-Slt2p above the untreated baseline level. Therefore, an association could be established between Ras2p expression, CWI pathway activation, and oxidative stress resistance.

The *wsc1*∆ strain was unable to accumulate P-Slt2p above the untreated baseline level when treated with 1mM H_2_O_2_ (Figure 7A) while the double mutant combination of *ras2*∆*wsc1*∆ caused a dramatic drop in P-Slt2p levels under the same treatment. These results indicated that the Ras2p-Wsc1p interaction is required for P-Slt2p accumulation in the response to oxidative stress and can be associated with CWI pathway activation. Similar to *wsc1*∆, the *ras2∆mid2∆* double mutant strains underwent reduced cell growth and decreased accumulation of P-Slt2p in response to 1 mM H_2_O_2_ (Figures 6C and 7A respectively), indicating that the physical interactions between Ras2p and Mid2p are important for resistance to oxidative stress and could be associated with CWI pathway activation. In control experiments, we excluded that H_2_O_2_ alone causes chemical interference with Slt2p phosphorylation because the wild-type control was able to accumulate P-Slt2p in the presence of 1mM H_2_O_2_ (Figure 7A).

It is known that Caspofungin inhibits β-1,3-glucan synthase. We therefore observed that upon exposure to 75 ng/ml Caspofungin, calibrated as the IC50 for this drug on agar cultures, the wild-type strain accumulated P-Slt2p above the untreated baseline level (Figure 7B). Upon treatment with 75 ng/ml Caspofungin, the *mid2∆*, *wsc1∆*, *zeo1∆*, and *ras2∆* single mutant strains all accumulated P-Slt2p at levels similar to the wild-type control strain (Figure 7B). Only the double mutant strains *ras2∆mid2∆* and *ras2∆wsc1∆* were unable to accumulate P-Slt2p levels in response to Caspofungin treatment (Figure 7B). These observations correlated with their inability to grow on agar cultures treated with 75 ng/ml Caspofungin. Thus, the physical interactions between Ras2p-Mid2p and Ras2p-Wsc1p were important for resistance to cell wall stress by Caspofungin and could be associated with CWI pathway activation.

## Discussion

The purpose of this study was to identify novel interacting protein partners of yeast transmembrane stress receptor proteins Wsc1 and Mid2, and to determine if they are required for survival under specific stress conditions, or be functionally associated to CWI signaling. We used the iMYTH technique to identify putative novel interacting protein partners. Confirmatory iMYTH testing was performed. This resulted in the identification of 14 Wsc1p and 31 Mid2p interactors. Two interactors of Mid2p, namely Zeo1p (Green *et al.* 2003) and Pst2p (Tarassov *et al.* 2008) were previously reported in other studies and were revalidated in this iMYTH screen. These interacting proteins represented important biological functions that include stress response, cell wall organization, protein phosphorylation, signal transduction, protein targeting, cytoskeleton organization, DNA repair, protein regulation, ribosome metabolism, and protein transport as well four with previously unknown functions. Of particular interest for potential roles in regulating Wsc1p and Mid2p signaling were Ras2p, Zeo1p, and Yck1p because of their known localization at the plasma membrane compartment and their respective biological process categories related to signaling: “G-protein”, “adaptor protein”, and “protein kinase” respectively.

Validation of iMYTH interacting protein partners by alternative physical methods such as affinity purification (AP) or immunoprecipitation (IP) coupled with mass spectrometry or western blotting (Table 2, Table 3, Table 4 and Figure 4 respectively) resulted in a physical interactome of lower complexity with only 13 proteins compared to the initial combined iMYTH interactome of 34. Surprisingly, eight out of the 13 interactor proteins were shared between both sensors (Figure 5). This result provided supporting evidence for the functional redundancy that exists between Wsc1p and Mid2p, despite these being members of different protein families (Gray *et al.* 1997; Torres *et al.* 2002).

The classification of candidates among these 13 interactor proteins as components of putative stress signaling complexes was based on three general criteria: 1) their cellular localization at the plasma membrane compartment, 2) a biochemical function that may be associated with a signaling protein function such as: a protein kinase, a GTP binding protein, an adaptor protein, a regulatory protein, and 3) functional genetic test results of null mutants that were consistent with a role in the stress response such as growth inhibition or failure to accumulate P-Slt2p, commonly used as an indirect measure of CWI pathway activation. By these criteria, we identified components of two putative signaling complexes defined by plasma membrane localization, biochemical function(s), and protein-protein interactions identified in null deletion strains with bait-prey combinations, that exhibited the strongest growth sensitivity to stress conditions. Two four-protein complexes are proposed for H_2_O_2_-induced oxidative stress resistance (Figure 6A) and for Caspofungin-induced cell wall stress resistance respectively (Figure 6B).

At the core of the putative signaling complexes for both oxidative and cell wall stress are the signaling proteins Wsc1, Mid2 and Ras2. Although we expected that Wsc1p and Mid2p would participate in the activation of Pkc1p (Heinisch *et al.* 2010), they each shared Ras2p as an interactor. Ras2p has been previously described as a positive regulator of adenylate cyclase (Broek *et al.* 1985; Toda *et al.* 1985) and it is related to the CWI and TOR pathways through Rom2p (Park *et al.* 2005). Furthermore, Ras2p and Wsc1p have a genetic interaction under heat shock stress (Verna *et al.* 1997). However, our study is the first one demonstrating a physical interaction between Ras2p and Wsc1p or Mid2p. It is now evident from this study that Ras2p interacts physically with Wsc1p or Mid2p and that it can regulate the accumulation of P-Slt2p by a mechanism possibly related to CWI pathway although the participation of Ras2p in a cAMP-independent mechanism for stress response has not been dismissed (Shama *et al.* 1998). Although the mechanism was not deciphered in this study, these results provide circumstantial evidence for a crosstalk mechanism between Pkc1p and the Pkap complex mediated by Ras2p. Such a crosstalk mechanism was previously proposed by others for the stress sensor Mtl1p (a member of the Mid2p family) (Petkova *et al.* 2010).

Grx1p functions as a Glutathione-dependent disulfide oxido-reductase that protects cells from oxidative damage (Luikenhuis *et al.* 1998), while Atx1p is a cytosolic copper metallochaperone (Lin and Culotta 1995) required for resistance to oxidative damage by 1 mM H_2_O_2_. Neither of these proteins are associated with the plasma membrane compartment yet both were proven to interact physically with Mid2p, and were functionally associated with accumulation of P-Slt2 and cell survival under oxidative stress. Because they did not re-validate their iMYTH interaction by physical testing methods such as IP-MS, AP-MS, or AP-WB, we propose that these cytoplasmic proteins are likely to be transient interactors that share weak interactions with Mid2p as a putative oxidative stress signaling complex (Figure 5). The other Mid2p interactor specifically associated with Caspofungin stress signaling was Ras2p that also functions in the oxidative stress response mechanism discussed above. The Caspofungin stress response signaling complex also shares the Wsc1p-Ras2p-Mid2p interaction, with the addition of Zeo1p, which was previously identified as an interactor of Wsc1p and Mid2p (Philip and Levin 2001; Vay *et al.* 2004) as an adaptor protein for Rom2p.

Surprisingly, Rom2p was not identified in our iMYTH screens. Also, Wsc1p-Rom2p and Mid2p-Rom2p interactions were not validated by AP-WB using Wsc1p or Mid2p as baits or in an inverted format using Rom2p as bait and Wsc1p or Mid2p as preys in separate pull-down experiments. To explain the difficulty in detecting the putative Wsc1p-Rom2p and Mid2p-Rom2p complexes, we believe that the result may have been due to a low level of expression of the Rom2p-TAP fusion protein in the total protein extracts employed in the AP-WB experiments. Supporting this argument was the observation that Zeo1p, also reported to be an interactor of Mid2p (Saccharomyces Genome Database, www.yeastgenome.org), exhibited relatively high expression levels in total protein extracts, was identified with very high frequency in our iMYTH screens employing the same prey library, and was consistently re-confirmed by alternative protein-protein interaction assays. Furthermore, the iMYTH experimental design used in this study was not conducted as a saturation screen for Wsc1p or Mid2p interacting partners. Therefore, there may have been Rom2p prey clones sparsely represented in the prey cDNA library that were not selected by the random screening approach employed here.

Because Wsc1p and Mid2p were previously described to respond to thermal stress in a similar way (23), we also conducted an iMYTH screen for heat stress at 37°. A dramatic reduction in the complexity of the 30° iMYTH interactomes of Wsc1p and Mid2p was observed (Figure S9). We interpreted that the observed reduction in the number of interactions was due to the destabilization of the 30° interacting partners at the higher temperature, with only the most stable interactions remaining at 37°. Heat stress resulted in 2 iMYTH interactions for Wsc1p (Ade2p, an Phosphoribosylaminoimidazole carboxylase and Egd2p, the alpha subunit of the nascent polypeptide-associated complex (NAC), and 6 for Mid2p (Egd2p already described; Zeo1p, an adaptor protein for Rom2p; Ras2p, a small GTP binding protein; and ribosomal proteins Rpl11b, and Rps1b). The predominant interactor at 37° was Egd2p which was a shared between Wsc1p and Mid2p. While Ras2p, Rps1bp, and Rpl11bp knockout strains were previously reported to exhibit increased heat sensitivity in null mutant strains (Sinha *et al.* 2008; Ma *et al.* 2012), we did not observe temperature such sensitivity in our null mutant strains for any of these proteins nor were they required for accumulation P-Slt2p at 37° (data not shown). Therefore, no specific function could be ascribed to any of these Mid2p interactors in the heat stress response mechanism.

The Wsc1p interactors Ypl238cp and Pst2p, previously annotated in the SGD as “putative proteins of unknown function” were validated by confirmatory physical tests. Ypl238cp, is described as an integral component of the membrane for which there is no known biological function (www.yeastgenome.org). Pst2p, which also shares interaction with Mid2p, is described as a protein with similarity to a family of flavodoxin-like proteins; that is induced by oxidative stress (Grandori and Carey 1994; Lee *et al.* 1999; Aittamaa *et al.* 2001). Yet, we could not ascribe any specific function for Pst2p in the oxidative stress protection tests used in this study. Nonetheless, we are confident that the interaction of Ypl238c and Pst2p with Wsc1p and Mid2p has been convincingly confirmed by this study and that these new annotations will be added. It is an intruiging possibility that Yck1p, identified as a shared interactor of Wsc1p and Mid2p, could represent the phosphorylating kinase for these stress sensors. However, the existence of the paralog Yck2p with which it shares redundant functions, limited our ability to detect an effect of individual *yck1∆* and *yck2∆* deletions on the accumulation of P-Slt2p, while the *yck1∆yck2∆* double mutant is inviable. Future studies of Yck1p and Yck2p phosphoproteomes must be directed at testing Wsc1p or Mid2p as potential substrates.

Sequence conservation of the *S. cerevisiae* stress sensor Wsc1p and Mid2p is observed among medically and commercially important fungi such as *Kluyveromycs lactis* (Rodicio *et al.* 2008), *Aspergillus fumigatus* (Dichtl *et al.* 2012), *Candida albicans* and *Candida glabrata* (Candida Genome Database). It is to be expected that at least some of the new interactions detected in this work may thus also be conserved and provide possible targets for the development of antifungal drugs.
